# Supplementary material for: Association of miR-146a-5p and miR-21-5p with Prognostic Features in Melanomas
Source: Cancers (Basel). 2024 Apr 26;16(9):1688. doi: 10.3390/cancers16091688 (PMC11083009; doi:10.3390/cancers16091688)
Supplement: Supplementary file 1 [file cancers-16-01688-s001.zip › cancers-2925583-supplementary.pdf]

Supplementary Figure Legend:

Figure S1. Correlation analysis of combined and individual miRNA expression with Breslow thickness (BT) in nodular melanoma (NM) and in lentigo maligna melanoma (LMM) subtypes.

Figures show the distribution of paired combined and individual miRNA values and BT values. The correlation in NM (A) and in LMM (B) is not significant. Simple linear regression line is shown.

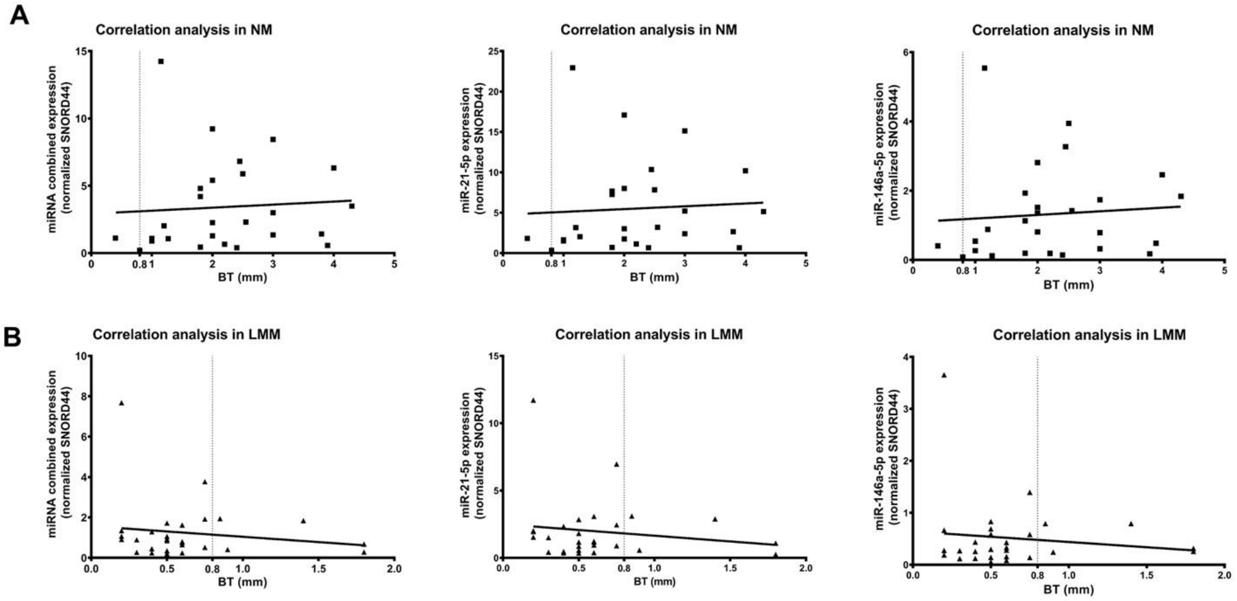

Figure S2. Association of combined and individual miR-21-5p and miR-146a-5p expression with prognostic parameters in CM histological subtypes

Figures show the scatter plot distribution of combined miRNA expression and individual miR-21-5p and miR-146a-5p in superficial spreading melanomas (SSM), nodular melanoma (NM) and lentigo maligna melanoma (LMM) based on Breslow thickness (BT) (A), ulceration status (B) and mitotic rate (C). Figures D show the scatter plot distribution of combined miRNA expression and individual miR-21-5p and miR-146a-5p in all melanomas (CM), SSM and NM based on regression status.

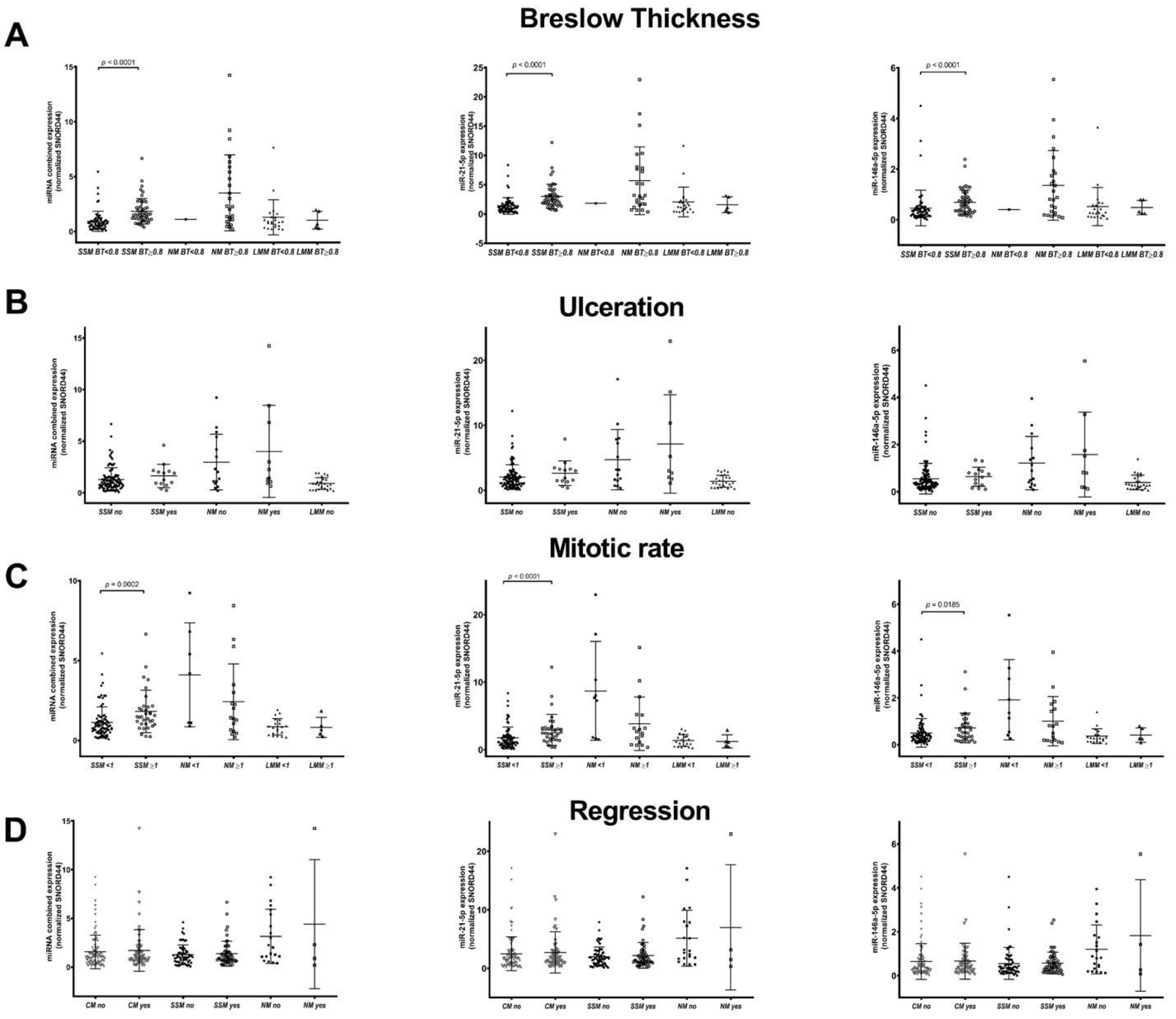

Figure S3. Kaplan-Meier plots for overall survival (OS) and time-to-relapse (TTR) in mitotic rate and regression groups.

(A) The plots show a significantly worse OS and TTR curves in patients with ≥1 mitosis/mm2 compared to patients with <1 mitosis/mm2. (B) The plots show no statistically different OS and TTR curves in patients grouped based on regression status

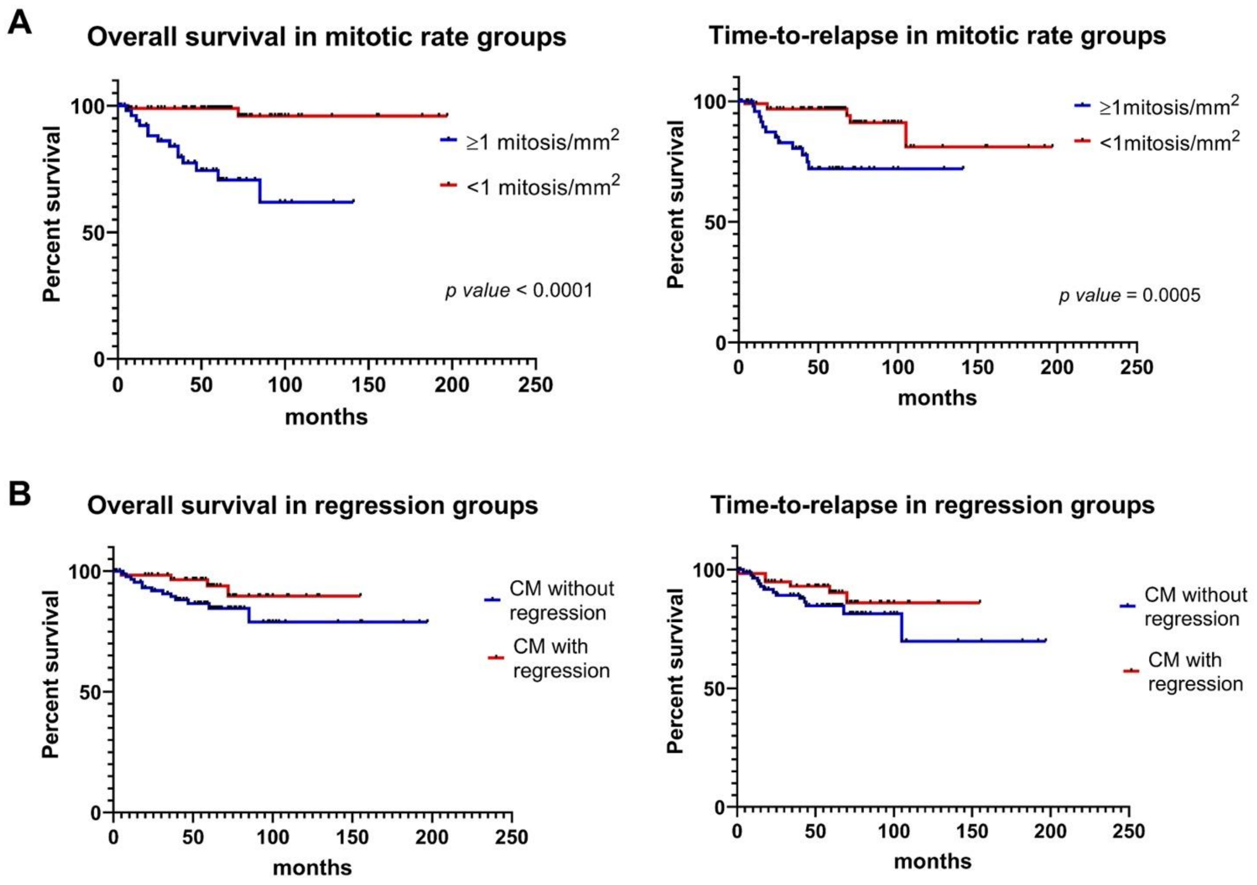

Breslow classification

T category	Breslow Thickness	Ulceration status
TX	N/A	N/A
Tis	N/A	N/A
T1	≤1.0 mm	Unknown or unspecified
T1a	<0.8 mm	Without ulceration
T1b	<0.8 mm	With ulceration
	0.8–1.0 mm	With or without ulceration
T2	>1.0–2.0 mm	Unknown or unspecified
T2a	>1.0–2.0 mm	Without ulceration
T2b	>1.0–2.0 mm	With ulceration
T3	>2.0–4.0 mm	Unknown or unspecified
T3a	>2.0–4.0 mm	Without ulceration
T3b	>2.0–4.0 mm	With ulceration

T4	>4.0 mm	Unknown or unspecified
T4a	>4.0 mm	Without ulceration
T4b	>4.0 mm	With ulceration

The original and primary source is the AJCC Cancer Staging Manual, 8th Edition (2017), published by Springer International Publishing (Gershenwald JE, Scolyer RA, Hess KR, et al. Melanoma of the Skin. In: Amin AB, Edge SB, Greene, FL, et al. (Eds). AJCC Cancer Staging Manual. 8th Ed. New York: Springer; 2017:563–585).

Clark levels of skin cancer.

In Clark Level I, the cancer is in the epidermis only.

In Clark Level II, the cancer has begun to spread into the papillary dermis (upper layer of the dermis).

In Clark Level III, the cancer has spread through the papillary dermis into the papillary-reticular dermal interface but not into the reticular dermis (lower layer of the dermis).

In Clark Level IV, the cancer has spread into the reticular dermis.

In Clark Level V, the cancer has spread into the subcutaneous tissue.
